# Supplementary material for: Atypical and Unique Transmission of Monkeypox Virus during the 2022 Outbreak: An Overview of the Current State of Knowledge
Source: Viruses. 2022 Sep 11;14(9):2012. doi: 10.3390/v14092012 (PMC9501469; doi:10.3390/v14092012)
Supplement: Supplementary file 1 [file viruses-14-02012-s001.zip › viruses-1877112-supplementary.pdf]

Search Criteria:

We reviewed PubMed and Google Scholar using search terms that included “monkeypox”, “monkeypox transmission”, “smallpox”, and “smallpox transmission” (up to August 29, 2022). Titles and abstracts of articles were first reviewed to determine whether a) the study was about monkeypox or smallpox and b) it addressed the current or a historical outbreak, transmission between animals, transmission between humans, spillover events, or epidemiological data. Our PubMed search yielded 12,620 studies across both the MPXV and the smallpox literature. 216 studies were then found to be potentially relevant to this review, of which 108 included here.

Supplemental Table S1. Case distribution. MPX cases and deaths per country described in the current outbreak through September 2, 2022. Blue shading indicates endemic countries.

Country	Cases	Deaths
Andorra	4	0
Argentina	170	0
Aruba	2	0
Australia	125	0
Austria	271	0
Bahamas	2	0
Barbados	1	0
Belgium	706	0
Benin	3	0
Bermuda	1	0
Bolivia	79	0
Bosnia and Herzegovina	3	0
Brazil	5037	1
Bulgaria	4	0
Cameroon	7	0
Canada	1251	0
Central African Republic	8	2
Chile	381	0
Colombia	582	0
Costa Rica	3	0
Croatia	26	0
Cuba	2	1
Cura√ßao	1	0
Cyprus	5	0
Czechia	48	0
Democratic Republic of the Congo	195	0
Denmark	175	0
Dominican Republic	7	0
Ecuador	53	1
El Salvador	1	0
Estonia	10	0
Finland	22	0
France	3558	0
Georgia	2	0
Germany	3493	0
Ghana	76	3
Gibraltar	6	0
Greece	58	0
Greenland	2	0
Guadeloupe	1	0
Guatemala	8	0
Guyana	2	0
Honduras	4	0
Hungary	70	0
Iceland	12	0
India	10	1
Indonesia	1	0
Iran	1	0
Ireland	144	0
Israel	239	0

Italy	760	0
Jamaica	5	0
Japan	4	0
Latvia	4	0
Lebanon	6	0
Liberia	2	0
Lithuania	5	0
Luxembourg	53	0
Malta	31	0
Martinique	1	0
Mexico	504	0
Moldova	2	0
Monaco	3	0
Montenegro	2	0
Morocco	3	0
Netherlands	1166	0
New Caledonia	1	0
New Zealand	4	0
Nigeria	220	4
Norway	82	0
Panama	10	0
Paraguay	1	0
Peru	1531	0
Philippines	4	0
Poland	130	0
Portugal	871	0
Qatar	3	0
Republic of the Congo	3	0
Romania	36	0
Russia	1	0
Saint Martin	1	0
Saudi Arabia	8	0
Serbia	31	0
Singapore	16	0
Slovakia	12	0
Slovenia	43	0
South Africa	5	0
South Korea	1	0
Spain	6543	2
Sudan	2	0
Sweden	161	0
Switzerland	476	0
Taiwan	3	0
Thailand	7	0
Turkey	1	0
United Arab Emirates	16	0
United Kingdom	3413	0
United States	19961	0
Uruguay	4	0
Venezuela	3	0
